# MicroRNA PC-3p-2869 Regulates Antler Growth and Inhibits Proliferation and Migration of Human Osteosarcoma and Chondrosarcoma Cells by Targeting CDK8, EEF1A1, and NTN1

**DOI:** 10.3390/ijms241310840

**Published:** 2023-06-29

**Authors:** Fan Yang, Jin Wu, Mindie Zhao, Han Zheng, Jingyuan Suo, Xuedong Liu, Dong Zheng

**Affiliations:** 1 Laboratory of Genetics and Molecular Biology, College of Wildlife and Protected Area, Northeast Forestry University, Harbin 150040, China; fanyang@nefu.edu.cn (F.Y.); nefu_wujin189@outlook.com (J.W.); 2020207070@stu.njau.edu.cn (M.Z.); suojy@nefu.edu.cn (J.S.); 2 Biotechnology Program, Division of Biology and Medicine, Brown University, Providence, RI 02912, USA; han_zheng2@brown.edu

**Keywords:** antler, osteosarcoma, chondrosarcoma, microRNA PC-3p-2869, cell proliferation, cross-species regulation

## Abstract

MicroRNAs (miRNAs) play a crucial role in maintaining the balance between the rapid growth and suppression of tumorigenesis during antler regeneration. This study investigated the role of a novel miRNA, PC-3p-2869 (miR-PC-2869), in antler growth and its therapeutic potential in human osteosarcoma and chondrosarcoma. Stem-loop RT-qPCR showed that miR-PC-2869 was expressed extensively in diverse layers of antler tissues. Overexpression of miR-PC-2869 suppressed the proliferation and migration of antler cartilage cells. Similarly, heterologous expression of miR-PC-2869 reduced the proliferation, colony formation, and migration of osteosarcoma cell line MG63 and U2OS and chondrosarcoma cell line SW1353. Moreover, 18 functional target genes of miR-PC-2869 in humans were identified based on the screening of the reporter library. Among them, 15 target genes, including CDK8, EEF1A1, and NTN1, possess conserved miR-PC-2869-binding sites between humans and red deer (*Cervus elaphus*). In line with this, miR-PC-2869 overexpression decreased the expression levels of CDK8, EEF1A1, and NTN1 in MG63, SW1353, and antler cartilage cells. As expected, the knockdown of CDK8, EEF1A1, or NTN1 inhibited the proliferation and migration of MG63, SW1353, and antler cartilage cells, demonstrating similar suppressive effects as miR-PC-2869 overexpression. Furthermore, we observed that CDK8, EEF1A1, and NTN1 mediated the regulation of c-myc and cyclin D1 by miR-PC-2869 in MG63, SW1353, and antler cartilage cells. Overall, our work uncovered the cellular functions and underlying molecular mechanism of antler-derived miR-PC-2869, highlighting its potential as a therapeutic candidate for bone cancer.

## 1. Introduction

Deer antlers are unique organs capable of complete periodic regeneration in mammals; their growth is primarily driven by chondrogenesis and endochondral ossification [1]. Interestingly, gene expression profiles of antlers demonstrate a stronger correlation with osteosarcoma (r = 0.67–0.78) relative to normal bone tissue (r = 0.33–0.47) [2]. Despite exceeding the growth rate of cancer tissue at a remarkable 2.75 cm/day, antler growth remains precisely regulated and non-cancerous [3]. Recent studies using sequencing-based techniques have suggested that microRNA (miRNA)-mRNA regulatory networks may play an important role in antler growth and the prevention of antler tumorigenesis [4,5]. Thus, investigating miRNAs that display tumor-suppressive activity in antler growth presents new therapeutic opportunities for bone cancer.

Osteosarcoma and chondrosarcoma are the two most common types of primary malignant bone cancers. Osteosarcoma is most prevalent in adolescents and originates from malignant primitive mesenchymal cells that generate an osteoid matrix [6]. Conversely, chondrosarcoma is typically observed in adults aged 40–70 years and is characterized by the malignant synthesis of the cartilaginous matrix [7]. Osteosarcoma and chondrosarcoma account for less than 1% of all diagnosed cancers but are associated with significant morbidity and mortality [8]. As a result of advancements in surgical resection and chemotherapy, patients with low-grade bone cancer benefit from a 5-year survival rate of over 70% [8,9]. However, the prognosis is poor for patients with metastatic or dedifferentiated bone cancer [8]. Therefore, it is crucial to develop innovative therapeutic agents to improve the overall survival of patients with bone cancer.

MicroRNAs (miRNAs) are endogenous non-coding small RNA molecules approximately 19–24 nucleotides in length, which play critical regulatory roles in animals and plants by binding to complementary mRNAs for target cleavage or translational repression [10]. Insights into the roles of miRNAs in various physiological and pathological processes have made miRNAs attractive tools and targets for novel therapeutics [11,12]. Generally, a single miRNA simultaneously modulates numerous target genes, and in turn, the synergistic effects of target genes determine the biological function of miRNAs [13]. Exploration of the miRNA regulatory network facilitates understanding the exact molecular mechanism of miRNA and the potential applications of miRNA.

In our previous study, miRNA PC-3p-2869 (miR-PC-2869; GenBank: MW579529.1) was found as a novel miRNA by the sequencing of antler tissues [14]. Here, we demonstrated that miR-PC-2869 regulates the proliferation and migration of antler cartilage cells. Moreover, the heterologous expression of miR-PC-2869 inhibits the proliferation and migration of osteosarcoma and chondrosarcoma cells. In silico prediction and cell-based screening identified 18 direct targets of miR-PC-2869. Notably, cyclin-dependent kinase 8 (CDK8), eukaryotic translation elongation factor 1 alpha 1 (EEF1A1), and netrin 1 (NTN1), three functional targets of miR-PC-2869, jointly mediate the cellular functions of miR-PC-2869 in antler growth and bone cancers. In summary, this study demonstrated the cellular functions and underlying molecular mechanism of miR-PC-2869, providing valuable insight into the application of miR-PC-2869 for bone cancer.

## 2. Results

### 2.1. MiR-PC-2869 Is Widely Expressed in Antler Tissues and Regulates Antler Cell Proliferation

**Figure 1:**
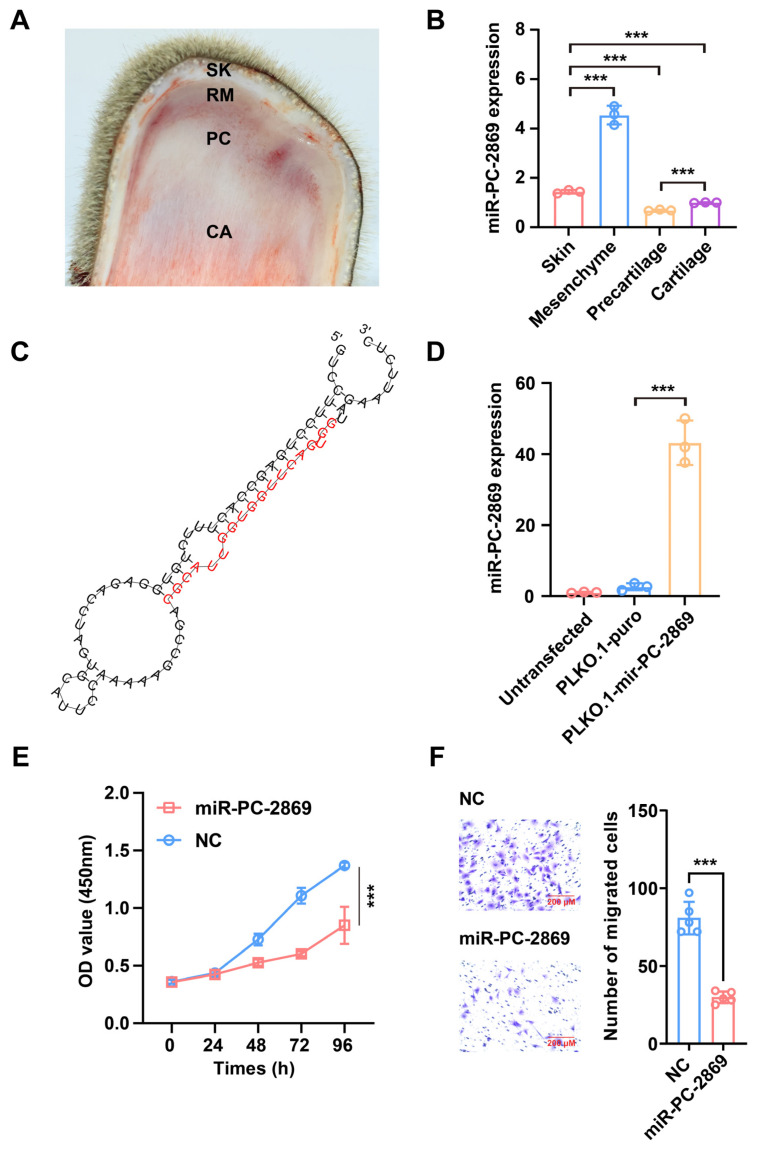
MiR-PC-2869 is widely expressed in antler tissues and regulates antler cartilage cell proliferation and migration. (**A**) Longitudinal section of antler growth tip, including skin (SK), reserve mesenchyme (RM), precartilage (PC), and cartilage (CA). (**B**) Relative expression levels of miR-PC-2869 in velvet antler tissues were analyzed by stem-loop RT-qPCR. (**C**) Schematic diagram of the structure of miR-PC-2869 precursor predicted by RNAfold web server. (**D**) Relative expression of mature miR-PC-2869 was assessed with stem-loop RT-qPCR in 293T cells transfected with PLKO.1-puro plasmid expressing miR-PC-2869 precursor (mir-PC-2869). (**E**,**F**) CCK-8 assay and transwell migration assay were performed to assess the proliferation (**E**) and migration (**F**) of antler cartilage cells, following transfection with miR-PC-2869 mimics and NC mimics. Scale bar, 200 μm. NC, negative control. *** *p* < 0.001.

To verify the presence of the miR-PC-2869 in deer antlers, we employed stem-loop RT-qPCR to detect miR-PC-2869 in various antler tissue layers (Figure 1A). As depicted in Figure 1B, the expression level of miR-PC-2869 decreased sequentially in the mesenchyme, skin, cartilage, and precartilage. The stem-loop structure of the miR-PC-2869 precursor was predicted using the RNAfold web server (Figure 1C). Transfecting the plasmid expressing miR-PC-2869 precursor into HEK293T cells significantly increased the expression level of mature miR-PC-2869 (Figure 1D). To investigate the role of miR-PC-2869 in antler growth, antler cartilage cells were transfected with miR-PC-2869 mimics or NC mimics. CCK-8 and transwell migration assays showed that overexpression of miR-PC-2869 significantly suppressed the proliferation and migration of antler cartilage cells (Figure 1E,F).

### 2.2. Heterologous Expression of miR-PC-2869 Inhibits the Proliferation and Migration of Osteosarcoma and Chondrosarcoma Cells

**Figure 2:**
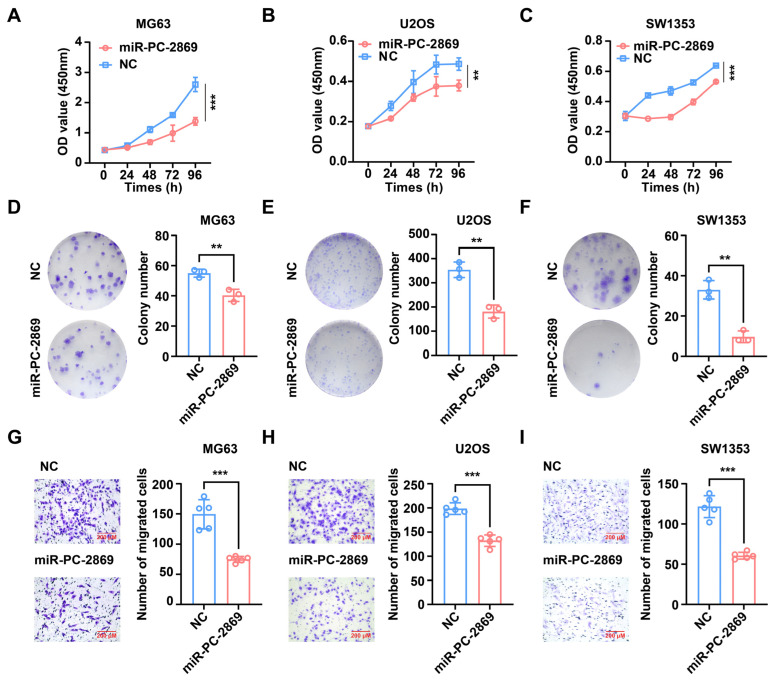
Heterologous expression of miR-PC-2869 inhibits the proliferation, colony formation, and migration of osteosarcoma and chondrosarcoma cells. MG63, U2OS, and SW1353 cells were transfected with miR-PC-2869 mimics or NC mimics. (**A**–**F**) CCK-8 and colony formation assays were conducted to evaluate the proliferative (**A**–**C**) and colony formation (**D**–**F**) capacities of the cells. (**G**–**I**) Migration of MG63 (**G**), U2OS (**H**), and SW1353 (**I**) cells was determined using transwell migration assay. Scale bar represents 200 μm. NC, negative control. ** *p* < 0.01; *** *p* < 0.001.

To explore the therapeutic potential of miR-PC-2869 in osteosarcoma and chondrosarcoma, miR-PC-2869 or NC mimics were transfected into MG63, U2OS, and SW1353 cell lines. The effect of miR-PC-2869 on cell proliferation was assessed using the CCK-8 assay. Our results indicated that the heterologous expression of miR-PC-2869 significantly repressed cell proliferation in all three cell lines (Figure 2A–C). Consistently, the colony formation assay revealed that miR-PC-2869 markedly reduced the colony-forming ability of MG63, U2OS, and SW1353 cells (Figure 2D–F). Additionally, the transwell migration assay demonstrated that miR-PC-2869 overexpression significantly decreased the number of migrated MG63, U2OS, or SW1353 cells compared to the NC group (Figure 2G–I).

### 2.3. Screening of miR-PC-2869 Targets Using the Reporter Library

**Figure 3:**
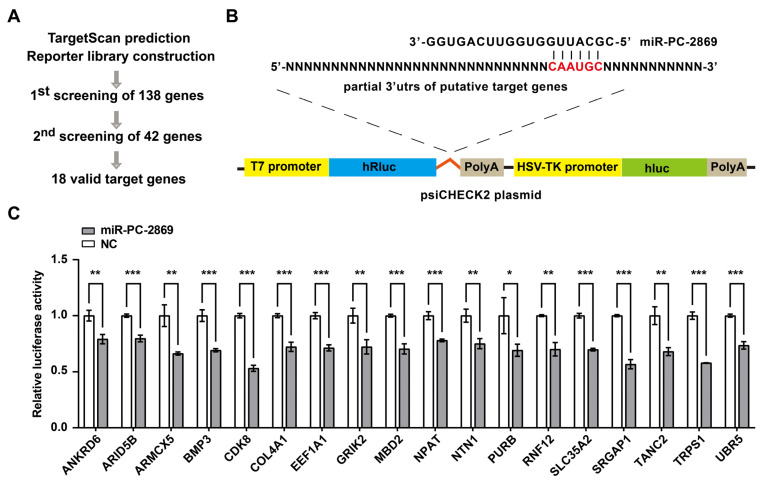
The reporter library screening determines target genes of miR-PC-2869. (**A**) The schematic diagram of the screening process for target genes. Initially, TargetScan predicted 138 potential target genes, and a reporter library was then constructed. Second, dual-luciferase assay of 138 reporters was performed independently twice, from which the top 42 genes with reproducible results were selected. Third, luciferase assays were performed in triplicates, and 18 target genes with statistically significant results were identified. (**B**) The schematic diagram illustrating the process of constructing luciferase reporters. (**C**) Luciferase assay of 18 reporters in HEK293T cells transfected with miR-PC-2869 mimics or NC mimics. NC, negative control. * *p* < 0.05; ** *p* < 0.01; *** *p* < 0.001.

To elucidate the molecular mechanism underlying the suppressive effects of miR-PC-2869 on cell proliferation and migration, we performed target gene prediction and screening analysis (Figure 3A). Using the TargetScan database (https://www.targetscan.org, accessed on 5 October 2021), and 138 human genes with putative target sites of miR-PC-2869 were identified. To validate these predicted target genes, fragments of 3′ UTRs containing wild-type miR-PC-2869-binding sites were cloned into dual-luciferase reporter plasmids to construct a reporter library (Figure 3B). After co-transfecting miR-PC-2869 or NC mimics and wild-type reporter constructs into HEK293T cells, the reporters of 18 putative target genes, i.e., ANKRD6, ARID5B, ARMCX5, BMP3, CDK8, COL4A1, EEF1A1, GRIK2, MBD2, NPAT, NTN1, PURB, RNF12, SLC35A2, SRGAP1, TANC2, TRPS1, and UBR5, were significantly downregulated by miR-PC-2869 (Figure 3C). Collectively, these results determined 18 target genes of miR-PC-2869.

### 2.4. miR-PC-2869 Directly Regulates 18 Target Genes

**Figure 4:**
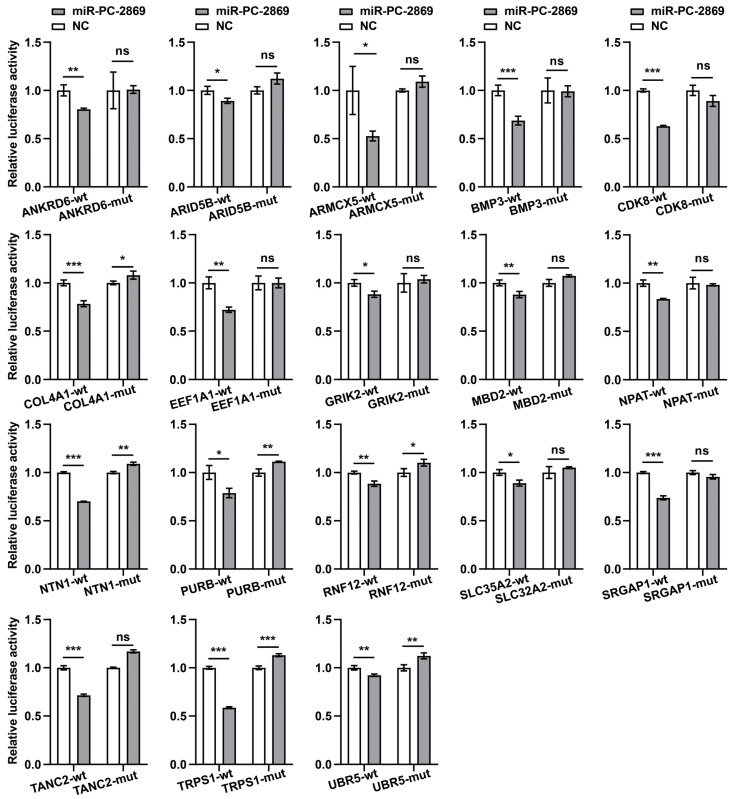
miR-PC-2869 directly targets specific sites in all 18 target genes. The luciferase activity was examined in HEK293T cells co-transfected with either wild-type (wt) or mutant (mut) reporter, along with miR-PC-2869 or negative control (NC) mimics. Renilla luciferase activity was normalized by Firefly luciferase activity. * *p* < 0.05; ** *p* < 0.01; *** *p* < 0.001; ns, not significant.

To further confirm whether miR-PC-2869 directly regulates these target genes, substitution mutations (sequence mutation from CAAUGC to AACUUC) were introduced into the miR-PC-2869-binding sites of 18 target genes. Subsequent dual-luciferase reporter assays revealed that overexpression of miR-PC-2869 significantly decreased the luciferase activity of the wild-type reporter constructs in HEK293T cells for all 18 target genes tested (Figure 4). However, miR-PC-2869 failed to inhibit the luciferase activity of mutated reporter constructs. These results indicate that miR-PC-2869 specifically recognizes these binding sites in 18 target genes.

### 2.5. Cross-Species Regulation of miR-PC-2869 on CDK8, EEF1A1, and NTN1

**Figure 5:**
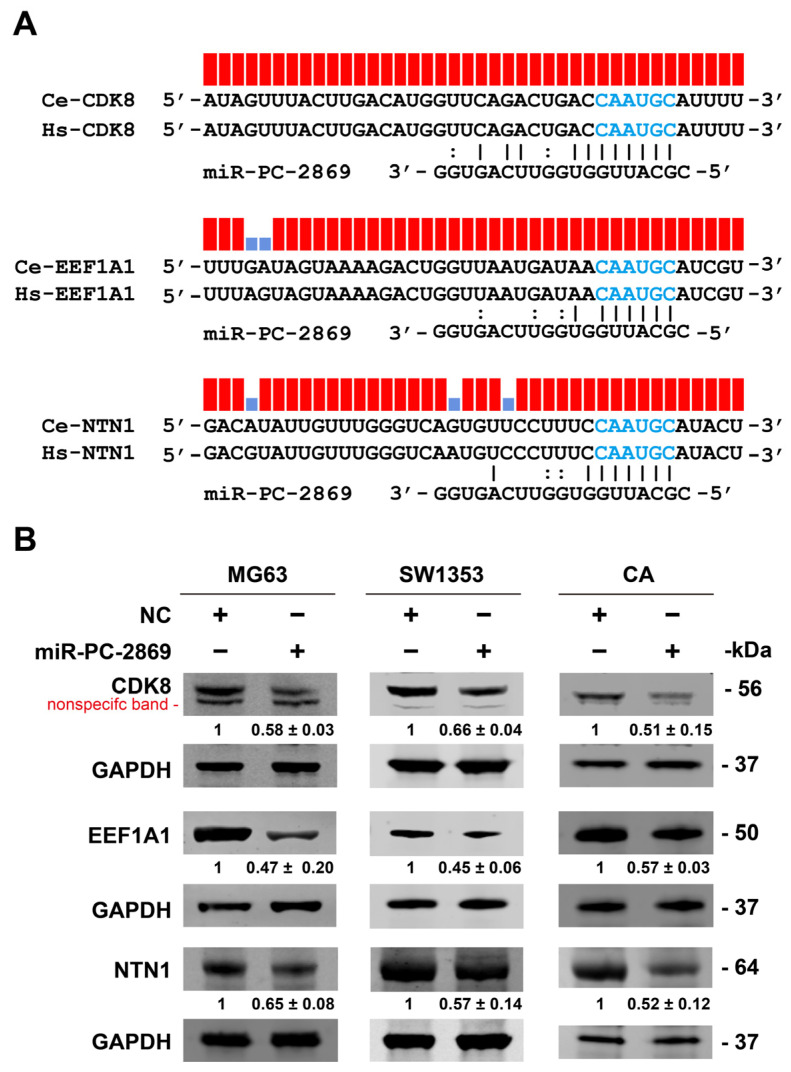
miR-PC-2869 regulates the expression of CDK8, EEF1A1, and NTN1. (**A**) The target sites of miR-PC-2869 in CDK8, EEF1A1, and NTN1 are conserved between humans and red deer (*Cervus elaphus*). Blue bases represent the complementary seed-matching region. (**B**) Western blot analysis of CDK8, EEF1A1, and NTN1 protein expression after transfection with miR-PC-2869 mimics or NC mimics in MG63, SW1353, and antler cartilage cells. The Western blot bands are representative images of three independent biological replicates, and fold-changes in densitometric values in three replicate experiments are presented as mean ± standard deviation. Ce, *Cervus elaphus*. Hs, *Homo sapiens*. NC, negative control. CA, antler cartilage cells.

Binding sites of miR-PC-2869 in 15 target genes, including CDK8, EEF1A1, and NTN1, are conserved between humans and red deer (*Cervus elaphus*; Figure 5A and Figure S1). Correspondingly, endogenous protein levels of CDK8, EEF1A1, and NTN1 were reduced in miR-PC-2869-overexpressing MG63, SW1353, and antler cartilage cells (Figure 5B).

### 2.6. The Knockdown of CDK8, EEF1A1, or NTN1 Results in Similar Inhibitory Effects on the Proliferation and Migration of Antler Cartilage Cells to the Overexpression of miR-PC-2869

**Figure 6:**
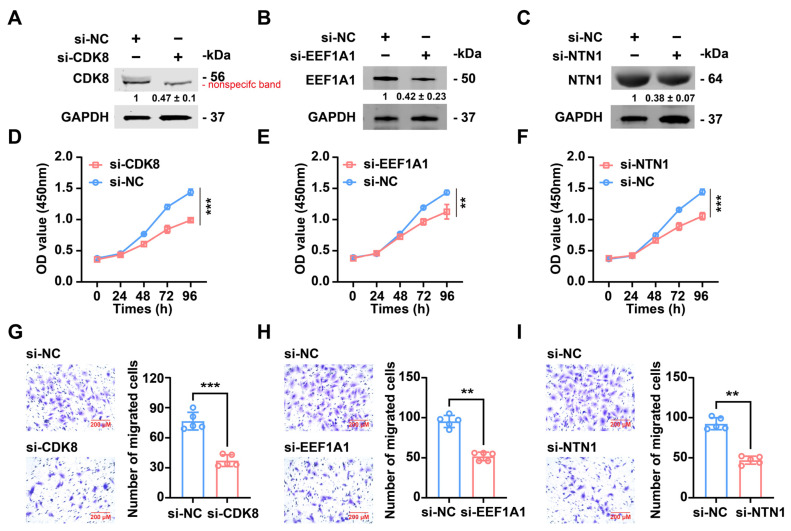
CDK8, EEF1A1, or NTN1 knockdown repressed the proliferation and migration of antler cartilage cells. (**A**–**C**) Western blot analysis of the interference efficiency of CDK8, EEF1A1, or NTN1 siRNA in antler cartilage cells. The Western blot bands are representative images of three independent biological replicates, and fold-changes in densitometric values in three replicate experiments are presented as mean ± standard deviation. (**D**–**F**) The proliferation of antler cartilage cells transfected with si-CDK8 (**D**), si-EEF1A1 (**E**), si-NTN1 (**F**), or si-NC was detected by CCK-8 assay. (**G**–**I**) The effect of si-CDK8 (**G**), si-EEF1A1 (**H**), or si-NTN1 (**I**) on antler cartilage cell migration was measured by transwell migration assays. Scale bar represents 200 μm. si-NC, negative control siRNA. ** *p* < 0.01, *** *p* < 0.001.

We investigated the functional targets of miR-PC-2869 in antler cartilage cells by examining the impact of CDK8, EEF1A1, and NTN1 knockdown on cellular proliferation and migration. The knockdown of each gene was successfully implemented using specific siRNAs, as evidenced by Figure 6A–C. Subsequently, we conducted the CCK-8 assay and observed a significant reduction in the proliferation of antler cartilage cells upon knockdown of CDK8, EEF1A1, or NTN1 compared to the si-NC group (Figure 6D–F). Moreover, the transwell migration assay demonstrated a drastic decrease in migrated antler cartilage cells upon siRNA-mediated silencing of CDK8, EEF1A1, or NTN1 (Figure 6G–I).

### 2.7. Silencing of CDK8, EEF1A1, or NTN1 Imitates the Tumor-Suppressor Activity of Heterologous miR-PC-2869 Expression in Osteosarcoma Cells

**Figure 7:**
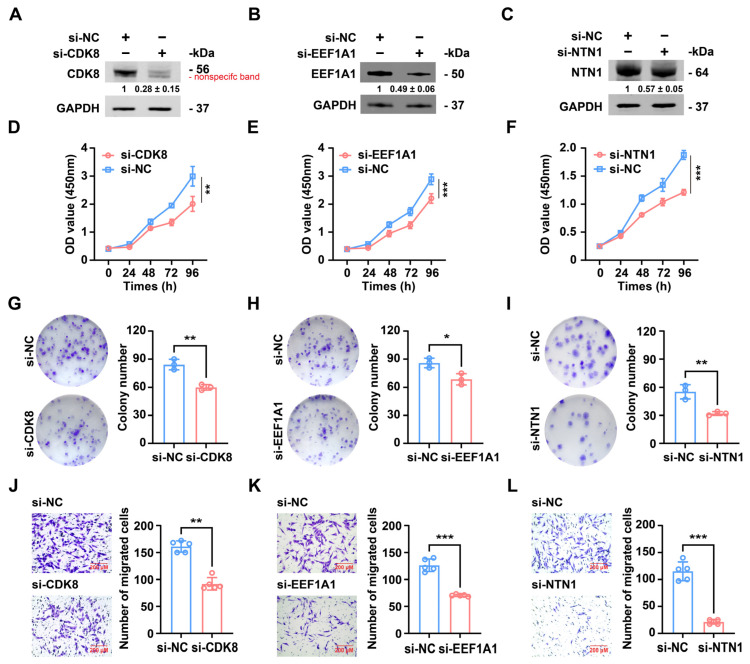
Downregulation of CDK8, EEF1A1, or NTN1 inhibits proliferation, colony formation, and migration of osteosarcoma cells. (**A**–**C**) Western blot analysis of MG63 cells transfected with CDK8 (**A**), EEF1A1 (**B**), or NTN1 (**C**) siRNA. The Western blot bands are representative images of three independent biological replicates, and fold-changes in densitometric values in three replicate experiments are presented as mean ± standard deviation. (**D**–**L**) Effects of CDK8, EEF1A1, or NTN1 knockdown on the proliferation (**D**–**F**), colony formation capacities (**G**–**I**), and migration (**J**–**L**) of MG63 cells were evaluated using CCK-8 assay, colony formation assay, and transwell migration assay, respectively. Scale bar represents 200 μm. si-NC, negative control siRNA. * *p* < 0.05; ** *p* < 0.01; *** *p* < 0.001.

CDK8, EEF1A1, and NTN1 have been confirmed as direct target genes of miR-PC-2869 in osteosarcoma cells, and their involvement in the pathogenesis of various human cancers has been previously reported. Thus, we hypothesized that CDK8, EEF1A1, and NTN1 mediate the tumor-suppressor activity of miR-PC-2869 in osteosarcoma cells. To test this hypothesis, specific siRNAs were applied to knock down the endogenous expression of CDK8, EEF1A1, and NTN1 in MG63 cells (Figure 7A–C). The CCK-8 proliferation assay showed that the knockdown of CDK8, EEF1A1, or NTN1 significantly inhibited the proliferation of MG63 cells compared to the control group (Figure 7D–F). Likewise, the suppression of CDK8, EEF1A1, or NTN1 led to a marked decrease in the ability of MG63 cells to form colonies, as shown by the colony formation assay (Figure 7G–I). Furthermore, the transwell migration assay revealed a noteworthy reduction in the number of migrated MG63 cells after the silencing of CDK8, EEF1A1, or NTN1 (Figure 7J–L).

### 2.8. Inhibition of CDK8, EEF1A1, or NTN1 Mimics the Tumor-Suppressive Effects of Heterologous miR-PC-2869 Expression in Chondrosarcoma Cells

**Figure 8:**
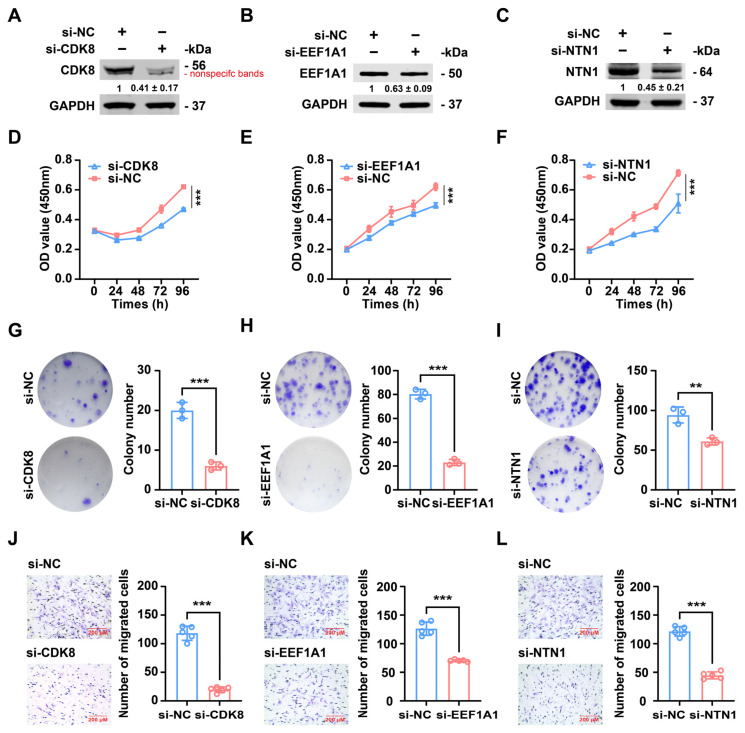
Silencing of CDK8, EEF1A1, or NTN1 suppressed the proliferation, colony formation, and migration of chondrosarcoma cells. (**A**–**C**) Western blot analysis of SW1353 cells transfected with si-CDK8, si-EEF1A1, si-NTN1, or si-NC. The Western blot bands are representative images of three independent biological replicates, and fold-changes in densitometric values in three replicate experiments are presented as mean ± standard deviation. (**D**–**L**) The effects of CDK8, EEF1A1, or NTN1 knockdown on the proliferation (**D**–**F**), colony formation capacities (**G**–**I**), and migration (**J**–**L**) of SW1353 cells were assessed by CCK-8 assay, colony formation assay, and transwell migration assay, respectively. Scale bar represents 200 μm. si-NC, negative control siRNA. ** *p* < 0.01; *** *p* < 0.001.

To assess whether miR-PC-2869 exerts inhibitory effects on chondrosarcoma cells by downregulating CDK8, EEF1A1, and NTN1, we employed RNA interference to individually knock down the expression of each gene in SW1353 cells. The knockdown efficacy was confirmed by Western blot analysis, which showed a significant reduction in the expression of CDK8, EEF1A1, or NTN1 in SW1353 cells, as depicted in Figure 8A–C. The CCK-8 assay indicated a substantial reduction in cell viability for the group with CDK8, EEF1A1, or NTN1 knockdown compared to the control group (Figure 8D–F). In the colony formation assay, the number of colonies formed by SW1353 cells was significantly reduced when CDK8, EEF1A1, or NTN1 expression was silenced (Figure 8G–I). Moreover, the migratory capability of SW1353 cells was severely inhibited following the downregulation of CDK8, EEF1A1, or NTN1 expression (Figure 8J–L).

### 2.9. MiR-PC-2869 Represses c-Myc and Cyclin D1 Expression by Targeting CDK8, EEF1A1 and NTN1

**Figure 9:**
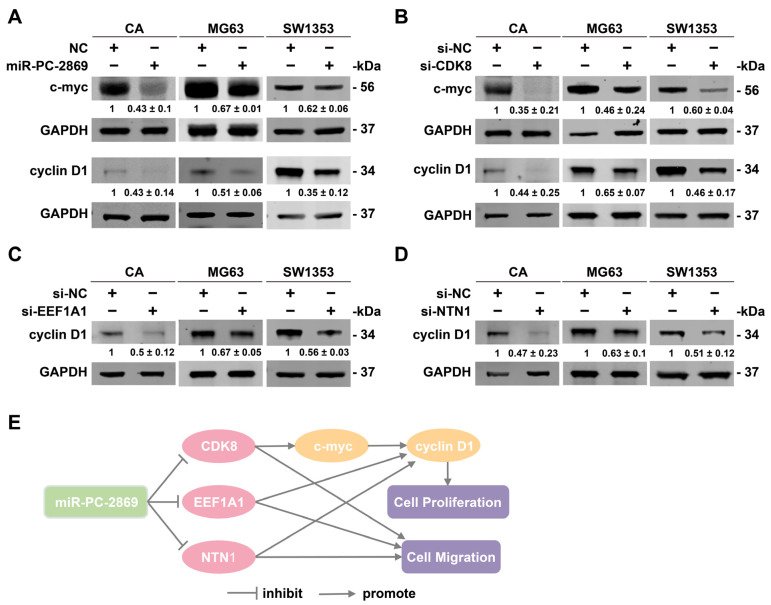
MiR-PC-2869 blocks the expression of c-myc and cyclin D1 by targeting CDK8, EEF1A1, and NTN1. (**A**–**D**) Western blot analysis of MG63, SW1353, and antler cartilage cells transfected with miR-PC-2869 mimics (**A**), si-CDK8 (**B**), si-EEF1A1 (**C**), or si-NTN1 (**D**). The Western blot bands are representative images of three independent biological replicates, and fold-changes in densitometric values in three replicate experiments are presented as mean ± standard deviation. NC, negative control mimics. si-NC, negative control siRNA. CA, antler cartilage cells. (**E**) The schematic diagram illustrates the molecular mechanism through which miR-PC-2869 inhibits the proliferation and migration of osteosarcoma, chondrosarcoma, and antler cartilage cells.

C-myc and cyclin D1 are vital regulators of cell cycle progression. Our results indicated that miR-PC-2869 overexpression significantly repressed their expression in MG63, SW1353, and antler cartilage cells (Figure 9A). It has been reported that CDK8, EEF1A1, and NTN1 facilitate cell proliferation by accelerating the G1/S transition of the cell cycle [15,16,17]. According to our observation, CDK8 knockdown reduced the expression of both c-myc and cyclin D1 in MG63, SW1353, and antler cartilage cells (Figure 9B), whereas silencing EEF1A1 or NTN1 suppressed cyclin D1 expression in all three cell types (Figure 9C,D).

## 3. Discussion

miRNAs are essential in preserving the balance between rapid growth and suppression of carcinogenesis during antler regeneration [4,5]. Our study confirmed the existence of a novel miRNA, miR-PC-2869, in antlers and further revealed its ability to inhibit antler cartilage cell proliferation and migration. Remarkably, a gradual increase in proliferation index was observed in the cartilage, skin, and reserve mesenchyme of the antler growth center [18,19], consistent with the expression pattern of miR-PC-2869 in antler tissues. Therefore, miR-PC-2869 may regulate antler growth by preventing uncontrolled cellular proliferation.

Antlers have been recognized as a valuable source for discovering bioactive agents for cancer therapy, with previous studies mainly focusing on antler polypeptides and extracts [20,21]. Our recent work [12] and the current study demonstrated the therapeutic potential of small nucleic acid molecules derived from antlers in cancer cells. Indeed, cross-species application of animal or plant miRNAs in human cancer has been widely reported [22,23,24]. For example, shrimp miR-S8 and miR-35 suppressed the stemness of human melanoma stem-like cells. The biological function of miRNAs is reliant on their downstream targets. In this investigation, 18 functional target genes of miR-PC-2869 were confirmed. Among them, CDK8 [25], COL4A1 [26], EEF1A1 [27], GRIK2 [28], NTN1 [29], RNF12 [30], SLC35A2 [31], SRGAP1 [32], TRPS1 [33], and UBR5 [34] have been identified as drivers of cancer progression in other studies, implying the potential tumor-suppressive role of miR-PC-2869. In addition, specific sites recognized by miR-PC-2869 on 15 target genes are conserved between human and red deer, including CDK8, EEF1A1, and NTN1. As expected, miR-PC-2869 regulates the expression of CDK8, EEF1A1, and NTN1 in both human bone cancer and deer antler cells, suggesting that conserved target sites underlie the cross-species regulation of miR-PC-2869.

The CDK8 submodule mediates the transcriptional regulation of various signaling pathways, including TGFβ/BMP signaling and Wnt/β-catenin signaling, which are linked to cell proliferation, differentiation, and stemness [35]. Our findings indicated that repression of CDK8 led to a significant reduction in the proliferation, colony formation, and migration of osteosarcoma, chondrosarcoma, and antler cartilage cells. Additionally, the silencing of CDK8 induced downregulation of the oncogenes c-myc and cyclin D1 in these three cells. It is well known that cyclin D1, as a downstream gene of c-myc, regulates the G1/S cell cycle transition. Nevertheless, the regulatory mechanism between CDK8 and c-myc is complex and context-dependent. In colon cancer, CDK8 enhances the activity of β-catenin and suppresses the activity of E2F1 (repressor of β-catenin), thereby indirectly promoting the expression of c-myc [25,36]. However, CDK8 only alters the posttranslational modification landscape of c-myc protein in embryonic stem cells, thereby maintaining the stability of c-myc protein [37].

EEF1A1 binds actin and modulates the cytoskeleton organization responsible for cell migration [38,39]. Thus, it is plausible that the migratory ability of osteosarcoma, chondrosarcoma, and antler cartilage cells markedly declined with the inhibition of EEF1A1. In addition, EEF1A1 has been reported to control cell proliferation and cell cycle via activating MAPK signaling in colorectal cancer or STAT1 signaling in hepatocellular carcinoma [16,27]. Likewise, our findings indicate that the knockdown of EEF1A1 impedes cell proliferation and cyclin D1 expression in osteosarcoma, chondrosarcoma, and antler cartilage cells.

NTN1 is a pleiotropic secretory glycoprotein that functions as a ligand for two dependence receptors: deleted in colorectal cancer (DCC) and uncoordinated-5 homolog (UNC-5H). Accumulating evidence supports NTN1 and its dependence receptors as potential targets for cancer therapy [29,40,41,42,43]. Our observations showed that the suppression of NTN1 not only reduced the proliferation and migration of osteosarcoma, chondrosarcoma, and antler cartilage cells but also downregulated the expression of oncogenic cyclin D1 in these cells.

The knockdown of CDK8, EEF1A1, or NTN1 produces comparable results to miR-PC-2869 overexpression, indicating that these three genes may partially mediate the inhibitory effects of miR-PC-2869 on the c-myc/cyclin D1 signaling pathway and cell proliferation and migration (Figure 9E). However, further exploration is needed to understand the role of the other target genes in the miR-PC-2869 regulatory networks. Remarkably, the convergent cellular functions and molecular mechanisms of CDK8, EEF1A1, and NTN1 in antler cartilage cells, osteosarcoma cells, and chondrosarcoma cells support a similar genetic context between antler and bone cancer, as previously proposed by Wang et al. [2]. This finding provides another basis for the cross-species application of antler-derived miRNAs in bone cancer.

In conclusion, our study reveals the cellular function of miR-PC-2869 in antler growth, demonstrates the therapeutic potential of miR-PC-2869 in osteosarcoma and chondrosarcoma, elucidates the underlying molecular mechanisms of cross-species regulation of miR-PC-2869, and opens new avenues for the application of antler-derived miRNAs.

## 4. Materials and Methods

### 4.1. Tissue Collection and Cell Culture

Animal experiments in this study were conducted with the approval of the Animal Care, Use and Ethics Committee at Northeast Forestry University (approval no. UT-31; 20 June 2014). Antler tips of three healthy red deer (*Cervus elaphus*) were collected from the Qinhuangdao Wildlife Park in China, and the tissue layers were dissected from distal 5 cm of antler tips as described by Li et al. [44,45]. Cartilage tissue pieces were then digested with hyaluronidase (Solarbio, Beijing, China) for 1.5 h at 37 °C and collagenase-II (Solarbio) for 4 h at 37 °C. Antler cartilage cells were collected and cultured in high-glucose Dulbecco’s modified Eagle medium (DMEM) supplemented with 10% fetal bovine serum (FBS; Gibco, Grand Island, NY, USA), 200 U/mL penicillin, and 100 U/mL streptomycin at 37 °C with 5% CO_2_. Human embryonic kidney cell line HEK293T and human osteosarcoma cell lines U2OS and MG63 (Procell Life Science & Technology, Wuhan, China) were cultured in DMEM supplemented with 10% FBS at 37 °C with 5% CO_2_. Human chondrosarcoma cell line SW1353 (Procell Life Science & Technology) was cultured in Leibovitz’s L-15 medium (Gibco) containing 10% FBS at 37 °C in a humidified incubator with atmospheric air.

### 4.2. Quantitative Real-Time PCR

Total RNA was extracted from the antler tissues using the GeneJET RNA Purification Kit (Thermo Fisher Scientific, Waltham, MA, USA). The complementary DNA of miR-PC-2869 was synthesized with the miRNA 1st Strand cDNA Synthesis Kit (Vazyme, Nanjing, China). The expression of miR-PC-2869 was quantified using miRNA Universal SYBR qPCR Master Mix (Vazyme), while snRNA U6 was used as an internal control. The relative expression levels were calculated using the 2^−ΔΔCt^ method. The specific primers for miR-PC-2869 and U6 are provided in Table S1.

### 4.3. Plasmids, miRNA Mimics, and siRNAs

The miR-PC-2869 precursor was predicted using the RNAfold web server and subsequently synthesized by RuiBiotech (Harbin, China) before being cloned into the PLKO.1-puro plasmid (Miaolingbio, Wuhan, China). Candidate target genes of miR-PC-2869 were predicted using TargetScan databases 5.3 [46]. Based on these predictions, partial 3′ UTR sequences of the human target genes, which contains putative miR-PC-2869-binding sites, were synthesized and cloned into the psiCHECK2 plasmid, thereby constructing a reporter library (Promega, Madison, WI, USA). Additionally, reporter plasmids with the mutated miR-PC-2869 match sites were obtained by sequence synthesis and cloning. The miR-PC-2869 mimics, negative control mimics (NC), siRNA, and siRNA negative control (si-NC) were purchased from Synbio Technologies (Suzhou, China). The information on the synthetic sequences is shown in Tables S2 and S3.

### 4.4. Cell Transfection

DNA plasmids, miRNA mimics, or siRNAs were transfected into cells with jetPRIME^®^ DNA & siRNA Transfection Reagent (Polypus transfection, Illkirch, France) according to the manufacturer’s instructions. Cells were harvested at 24 h post-transfection and analyzed for further experimentation.

### 4.5. Cell Proliferation Assay

To assess cell proliferation capacity, transfected cells were seeded into 96-well plates at a density of 3000 cells per well and incubated for 24, 48, 72, or 96 h. Subsequently, cell counting kit-8 (CCK-8; Bimake, Houston, TX, USA) was added to each well and incubated at 37 °C for 2 h. The optical density was measured at 450 nm using the FilterMax F5 microplate reader (Molecular Devices, San Jose, CA, USA). The experiment was repeated three times.

### 4.6. Transwell Migration Assay

Cell migration was evaluated with Transwell Permeable Supports with an 8 μm pore size (Corning, Shanghai, China; Jet Biofil, Guangzhou, China). In brief, 5 × 10^4^ cells in 200 μL of serum-free media were added into the upper chamber, while the lower chamber was filled with 600 μL of medium containing 10% FBS. After incubation for 24 h, the non-migrated cells remaining on the upper surface of the membrane were removed. The migrated cells adhered to the lower membrane were fixed in 4% paraformaldehyde for 15 min, stained with 0.5% crystal violet for 15 min, and examined under the EVOS XL light microscope (Thermo Fisher Scientific) at a magnification of ×200. The experiment was repeated three times.

### 4.7. Colony Formation Assay

Single cells were seeded into six-well plates at a density of 400 cells per well for MG63 and SW1353 cells, or 1000 cells per well for U2OS cells. Following two weeks of incubation, cells were washed with PBS, fixed with paraformaldehyde for 15 min, stained with 0.4% crystal violet for 15 min, and subsequently photographed. The number of colonies in each well was counted. The experiment was repeated three times.

### 4.8. Dual-Luciferase Reporter Assay

To identify target genes of miR-PC-2869, HEK293T cells cultured in 24-well plates were co-transfected with miR-PC-2869 mimics and reporter construct plasmids. After 36 h of transfection, the luciferase activity was detected using the Dual-Luciferase Reporter Gene Assay Kit (Beyotime, Shanghai, China). Firefly luciferase activity served as an internal reference for Renilla luciferase activity.

### 4.9. Western Blot

The transfected cells were lysed with lysis buffer (Abcam, Cambridge, UK) containing 1% PMSF and 1% protease inhibitor cocktail (APExBIO, Houston, TX, USA) to extract total protein. The concentration of total protein was determined using the detergent compatible Bradford protein assay kit (Beyotime). After the separation of equal amounts of protein (30 µg) on 4–12% Bis-Tris SDS-PAGE gels (Thermo Fisher Scientific), the proteins were transferred to nitrocellulose membranes (0.45 µm; Merck, Billerica, MA, USA). Following blocking with 5% skim milk for 1 h, the membranes were incubated overnight at 4 °C with specific primary antibodies against CDK8 (cat. no. A5548; 1:1000; ABclonal, Wuhan, China), EEF1A1 (cat. no. ab157455; 1:1000; Abcam), NTN1 (cat. no. A1500; 1:1000; ABclonal), GAPDH (cat. no. sc-47724; 1:3000; Santa Cruz Biotechnology, Dallas, TX, USA; or cat. no. AC033; 1:20,000; ABclonal), c-myc (cat. no. A5011; 1:1000; Bimake), or cyclin D1 (cat. no. A5035; 1:1000; Bimake). After washing the membranes three times with Tris-buffered saline containing 0.1% Tween 20, the membranes were further incubated with goat anti-rabbit or goat anti-mouse secondary antibodies (1:15,000; LI-COR, Lincoln, NE, USA) for 1 h at room temperature and visualized using the Odyssey CLx near-infrared fluorescence imaging system (LI-COR). GAPDH was used as a loading control. Densitometric analysis of Western blot bands was performed using Image Studio Ver 5.2 software (LI-COR). The experiment was repeated three times.

### 4.10. Statistical Analysis

GraphPad Prism 9.0 (GraphPad Software Inc., San Diego, CA, USA) was utilized for all the statistical analyses. The normality of the distribution within each group was assessed using the Shapiro–Wilk W test (*p*-value > 0.05). For data that exhibited a non-normal distribution, the Mann–Whitney U test was applied to compare the groups. In the case of two independent groups with normal distributions, the unpaired two-tailed Student’s *t*-test was utilized for comparison. All values were presented as mean ± standard deviation (SD). *p* < 0.05 was considered statistically significant.

## Data Availability

All data and materials supporting the conclusions are included in the main paper.

## Supplementary Materials

The following supporting information can be downloaded at: https://www.mdpi.com/article/10.3390/ijms241310840/s1.
