# Supplementary material for: MicroRNA PC-3p-2869 Regulates Antler Growth and Inhibits Proliferation and Migration of Human Osteosarcoma and Chondrosarcoma Cells by Targeting CDK8, EEF1A1, and NTN1
Source: Int J Mol Sci. 2023 Jun 29;24(13):10840. doi: 10.3390/ijms241310840 (PMC10341717; doi:10.3390/ijms241310840)
Supplement: Supplementary file 1 [file ijms-24-10840-s001.zip › ijms-2442376-supplementary.pdf]

Supplementary materials

Table S1. Primers used for RT-qPCR

Primer name	Sequence (5′-3′)
miR-PC-2869-RT	GTCGTATCCAGTGCAGGGTCCGAGGTATTCGCACTGGATACGACC CACTG
miR-PC-2869-F	GCGCGCATTGGTGGTT
miR-PC-2869-R	AGTGCAGGGTCCGAGGTATT
Hs-U6-F	TGGAACGATACAGAGAAGATTAGCA
Hs-U6-R	AACGCTTCACGAATTTGCGT
Ce-U6-F	CTCGCTTCGGCAGCACA
Ce-U6-R	AATGCTTCACGAATTTGCGT

miR-PC-2869-RT is the reverse transcription primer. Hs: *Homo sapiens*. Ce: *Cervus elaphus*.

Table S2. Synthetic sequences used for cloning

DNA Oligos	sequence (5′-3′)
mir-PC-3p-2869-F	ccggGTCCTTCCTGAGCCACTTTCTGTGGAGACCTAGTGCATTCCA AAAAGCCGACGCATTGGTGGTTCAGTGGTAGAATTCTCTTTTTg
mir-PC-3p-2869-R	aattcAAAAAGAGAATTCTACCACTGAACCACCAATGCGTCGGCTT TTTGGAATGCACTAGGTCTCCACAGAAAGTGGCTCAGGAAGGAC
ANKRD6-wt-F	tcgagAACAGGCTCAGTCAGCATCCTCACCCAGAGATGGCAACATC TATTAAGACCAATGCAATACCTTTTCATCTTCgc
ANKRD6-wt-R	ggccgcGAAGATGAAAAGGTATTGCATTGGTCTTAATAGATGTTGCC ATCTCTGGGTGAGGATGCTGACTGAGCCTGTTc
ARID5B-wt-F	tcgagCAGATGGCAAGGGATGCCCCTCTTTTTCATAAAACTCTCCAA GGTTCAATCAATGCAATGTATAGTGAAACTTgc
ARID5B-wt-R	ggccgcAAGTTTCACTATACATTGCATTGATTGAACCTTGGAGAGTTT TATGAAAAAGAGGGGCATCCCTTGCCATCTGc
ARMCX5-wt-F	tcgagCTCTGTTCTCATAAAGCCTCAAACAGTTTTTTGGAGTTGCAA TATGAAACCAATGCATATTGTAATTATAAATgc

DNA Oligos	sequence (5′-3′)
ARMCX5-wt-R	ggccgcATTTATAATTACAATATGCATTGGTTTCATATTGCAACTCCAA<br>AAAACTGTTTGAGGCTTTATGAGAACAGAGc
BMP3-wt-F	tcgagTTATTTTTATGGACTTCTTCCTGTTTTTTTTTTTTTTTTTTTTGC<br>ACTGCCAATGCATTTTGTTTCAAAAGATgc
BMP3-wt-R	ggccgcATCTTTTGAAACAAAATGCATTGGCAGTGCAAAAAAAAAA<br>AAAAAAAAAACAGGAAGAAGTCCATAAAAATAAc
CDK8-wt-F	tcgagTGTTGTGGATTTGCTACTTCCATAGTTTACTTGACATGGTTCA<br>GACTGACCAATGCATTTTTTTTCAGTGACAGgc
CDK8-wt-R	ggccgcCTGTCACTGAAAAAAAATGCATTGGTCAGTCTGAACCATGTC<br>AAGTAAACTATGGAAGTAGCAAATCCACAACAc
COL4A1-wt-F	tcgagATACCAAATGGCACTTTTGATGAAATAAAATATCAATATTTTC<br>TGCAATCCAATGCACTGATGTGTGAAGTGAgc
COL4A1-wt-R	ggccgcTCACTTCACACATCAGTGCATTGGATTGCAGAAAATATTGAT<br>ATTTTATTTCATCAAAAGTGCCATTTGGTATc
EEF1A1-wt-F	tcgagCCATTTAAGTTTAGTAGTAAAAGACTGGTTAATGATAACAATG<br>CATCGTAAAACCTTCAGAAGGgc
EEF1A1-wt-R	ggccgcCCTTCTGAAGGTTTTACGATGCATTGTTATCATTAACCAGTC<br>TTTTACTACTAAACTTAAATGGc
GRIK2-wt-F	tcgagTTGACAACATTGTGATAACAGTTGAGTGCACACAGTTTGCTG<br>TTTGAATCCAATGCACAAAATTAAAAAAAAATgc
GRIK2-wt-R	ggccgcATTTTTTTTTAATTTTGTGCATTGGATTCAAACAGCAAACTGT<br>GTGCACTCAACTGTTATCACAATGTTGTCAAc
MBD2-wt-F	tcgagTGTAAGAAAAAAAAATGTACCCGAGCACATAGAGCTTTTTAAT<br>AGCACTAACCAATGCCTTTTTAGATGTATTTgc
MBD2-wt-R	ggccgcAAATACATCTAAAAAGGCATTGGTTAGTGCTATTAAAAAGC<br>TCTATGTGCTCGGGTACATTTTTTTTTCTTACAc
NPAT-wt-F	tcgagGGGATTTAAAGAATTGCACTAATTCATAGAACTCTTTACATTG<br>TAGTGACCAATGCACATATAATTTAAATCTgc

DNA Oligos	sequence (5′-3′)
NPAT-wt-R	ggccgcAGATTTAAATTATATGTGCATTGGTCACTACAATGTAAAGAGTTCTATGAATTAGTGCAATTCTTTAAATCCCc
NTN1-wt-F	tcgagTAAATGTTTAGTTGTGACCATGACGTATTGTTTGGGTCAATGTCCCTTTCCAATGCATACTAATATATTATGGgc
NTN1-wt-R	ggccgcCCATAATATATTAGTATGCATTGGAAAGGGACATTGACCCAAACAATACGTCATGGTCACAACTAAACATTTAc
PURB-wt-F	tcgagCTTCACTGTAAATTTTGTTTTCATTTTTAACCCAATTATGGTACTTTATCCAATGCACAACTGATCTCTCAGTgc
PURB-wt-R	ggccgcACTGAGAGATCAGTTGTGCATTGGATAAAGTACCATAATTGGGTTAAAAATGAAAACAAAATTTACAGTGAAGc
RNF12-wt-F	tcgagTTCATTTGTTTAAAAATGTAAAGCTATTTTGTAGAGGCTCAGTACTTTTCCAATGCACTGTTGTATGAATGCAgc
RNF12-wt-R	ggccgcTGCATTCATACAACAGTGCATTGGAAAAGTACTGAGCCTCTACAAAATAGCTTTACATTTTTAAACAAATGAAc
SLC35A2-wt-F	tcgagATTTTTTGGTTTGTTTTTTAAGGAACTGTAATGAACAAATGTCAGGATATCCAATGCCAAATAAAGATGTTGTgc
SLC35A2-wt-R	ggccgcACAACATCTTTATTTGGCATTGGATATCCTGACATTTGTTCATTACAGTTCCTTAAAAAACAAACCAAAAAATc
SRGAP1-wt-F	tcgagGACATATGTCATTTTAAGGACAATAGAAACACTTAGACTTACTTGAAAATCCAATGCTGCACCACTTGTAATGgc
SRGAP1-wt-R	ggccgcCATTACAAGTGGTGCAGCATTGGATTTTCAAGTAAGTCTAAGTGTTTCTATTGTCCTTAAAATGACATATGTCc
TANC2-wt-F	tcgagGGTTTTCTATGACTCAGATGTAAAGGACTTTCTCTGTACAGTATATTATCCAATGCATGTTTGTTCTCTCTCCgc
TANC2-wt-R	ggccgcGGAGAGAGAACAAACATGCATTGGATAATATACTGTACAGAGAAAGTCCTTTACATCTGAGTCATAGAAAACCc
TRPS1-wt-F	tcgagGTAGCACATTATGTTTATAATTGCACATGTGCACATAATCTATTATGATCCAATGCAAATACAGCTCCAAAAAgc

DNA Oligos	sequence (5′-3′)
TRPS1-wt-R	ggccgcTTTTTGGAGCTGTATTTGCATTGGATCATAATAGATTATGTGC<br>ACATGTGCAATTATAAACATAATGTGCTACc
UBR5-wt-F	tcgagGTAATTTGCTCATTTGAGTGAGGGCACTTTTTTTGTACATATG<br>ATGGGGCCAATGCACAATACTTTTATCACAgc
UBR5-wt-R	ggccgcTGTGATAAAAGTATTGTGCATTGGCCCCATCATATGTACAAA<br>AAAAGTGCCCTCACTCAAATGAGCAAATTACc
ANKRD6-mut-F	tcgagAACAGGCTCAGTCAGCATCCTCACCCAGAGATGGCAACATC<br>TATTAAGACAACTTCAATACCTTTTCATCTTCgc
ANKRD6-mut-R	ggccgcGAAGATGAAAAGGTATTGAAGTTGTCTTAATAGATGTTGCC<br>ATCTCTGGGTGAGGATGCTGACTGAGCCTGTTc
ARID5B-mut-F	tcgagCAGATGGCAAGGGATGCCCCTCTTTTTCATAAAACTCTCCAA<br>GGTTCAATAACTTCAATGTATAGTGAAACTTgc
ARID5B-mut-R	ggccgcAAGTTTCACTATACATTGAAGTTATTGAACCTTGGAGAGTTT<br>TATGAAAAAGAGGGGCATCCCTTGCCATCTGc
ARMCX5-mut-F	tcgagCTCTGTTCTCATAAAGCCTCAAACAGTTTTTTGGAGTTGCAA<br>TATGAAACAACTTCATATTGTAATTATAAATgc
ARMCX5-mut-R	ggccgcATTTATAATTACAATATGAAGTTGTTTCATATTGCAACTCCAA<br>AAAACTGTTTGAGGCTTTATGAGAACAGAGc
BMP3-mut-F	tcgagTTATTTTTATGGACTTCTTCCTGTTTTTTTTTTTTTTTTTTTGC<br>ACTGCAACTTCATTTTGTTTCAAAAGATgc
BMP3-mut-R	ggccgcATCTTTTGAAACAAAATGAAGTTGCAGTGCAAAAAAAAAA<br>AAAAAAAAACAGGAAGAAGTCCATAAAAATAAc
CDK8-mut-F	tcgagTGTTGTGGATTTGCTACTTCCATAGTTTACTTGACATGGTTCA<br>GACTGACAACTTCATTTTTTTCAGTGACAGgc
CDK8-mut-R	ggccgcCTGTCACTGAAAAAAATGAAGTTGTCAGTCTGAACCATGTC<br>AAGTAAACTATGGAAGTAGCAAATCCACAACAc
COL4A1-mut-F	tcgagATACCAAATGGCACTTTTGATGAAATAAAATATCAATATTTTC<br>TGCAATCAACTTCACTGATGTGTGAAGTGAgc

DNA Oligos	sequence (5′-3′)
COL4A1-mut-R	ggccgcTCACTTCACACATCAGTGAAGTTGATTGCAGAAAATATTGATATTTTATTTCATCAAAAGTGCCATTTGGTATc
EEF1A1-mut-F	tcgagCCATTTAAGTTTAGTAGTAAAAGACTGGTTAATGATAAATCGTAAAACCTTCAGAAGGAgc
EEF1A1-mut-R	ggccgcCCTTCTGAAGGTTTTACGATTTATCATTAACCAGTCTTTTACTACTAAACTTAAATGGAc
GRIK2-mut-F	tcgagTTGACAACATTGTGATAACAGTTGAGTGCACACAGTTTGCTGTTTGAATCAACTTCACAAAATTAAAAAAAATgc
GRIK2-mut-R	ggccgcATTTTTTTTAATTTTGTGAAGTTGATTCAAACAGCAAACTGTGTGCACTCAACTGTTATCACAATGTTGTCAAc
MBD2-mut-F	tcgagTGTAAGAAAAAAAATGTACCCGAGCACATAGAGCTTTTTAATAGCACTAACAACTTCCTTTTTAGATGTATTTgc
MBD2-mut-R	ggccgcAAATACATCTAAAAAGGAAGTTGTTAGTGCTATTAAAAAGCTCTATGTGCTCGGGTACATTTTTTTTCTTACAc
NPAT-mut-F	tcgagGGGATTTAAAGAATTGCACTAATTCATAGAACTCTTTACATTGTAGTGACAACTTCACATATAATTTAAATCTgc
NPAT-mut-R	ggccgcAGATTTAAATTATATGTGAAGTTGTCACTACAATGTAAAGAGTTCTATGAATTAGTGCAATTCTTTAAATCCCc
NTN1-mut-F	tcgagTAAATGTTTAGTTGTGACCATGACGTATTGTTTGGGTCAATGTCCCTTTCAACTTCATACTAATATATTATGGgc
NTN1-mut-R	ggccgcCCATAATATATTAGTATGAAGTTGAAAGGGACATTGACCCAAACAATACGTCATGGTCACAACTAAACATTTAc
PURB-mut-F	tcgagCTTCACTGTAAATTTTGTTTTCATTTTTAACCCAATTATGGTACTTTATCAACTTCACAACTGATCTCTCAGTgc
PURB-mut-R	ggccgcACTGAGAGATCAGTTGTGAAGTTGATAAAGTACCATAATTGGGTTAAAAATGAAAACAAAATTTACAGTGAAGc
RNF12-mut-F	tcgagTTCATTTGTTTAAAAATGTAAAGCTATTTTGTAGAGGCTCAGTACTTTTCAACTTCACTGTTGTATGAATGCAgc

DNA Oligos	sequence (5′-3′)
RNF12-mut-R	ggccgcTGCATTCATACAACAGTGAAGTTGAAAAGTACTGAGCCTCT ACAAAATAGCTTTACATTTTTAAACAAATGAAc
SLC35A2-mut-F	tcgagATTTTTTGGTTTGTTTTTTAAGGAACTGTAATGAACAAATGTC AGGATATCAACTTCCAAATAAAGATGTTGTgc
SLC35A2-mut-R	ggccgcACAACATCTTTATTTGGAAGTTGATATCCTGACATTTGTTCA TTACAGTTCCTTAAAAAACAAACCAAAAAATc
SRGAP1-mut-F	tcgagGACATATGTCATTTTAAGGACAATAGAAACACTTAGACTTACT TGAAAATCAACTTCTGCACCACTTGTAATGgc
SRGAP1-mut-R	ggccgcCATTACAAGTGGTGCAGAAGTTGATTTTCAAGTAAGTCTAA GTGTTTCTATTGTCCTTAAAATGACATATGTCc
TANC2-mut-F	tcgagGGTTTTCTATGACTCAGATGTAAAGGACTTTCTCTGTACAGTA TATTATCAACTTCATGTTTGTTCTCTCTCCgc
TANC2-mut-R	ggccgcGGAGAGAGAACAAACATGAAGTTGATAATATACTGTACAGA GAAAGTCCTTTACATCTGAGTCATAGAAAACCc
TRPS1-mut-F	tcgagGTAGCACATTATGTTTATAATTGCACATGTGCACATAATCTATT ATGATCAACTTCAAATACAGCTCCAAAAAgc
TRPS1-mut-R	ggccgcTTTTTGGAGCTGTATTTGAAGTTGATCATAATAGATTATGTG CACATGTGCAATTATAAACATAATGTGCTACc
UBR5-mut-F	tcgagGTAATTTGCTCATTTGAGTGAGGGCACTTTTTTTGTACATATG ATGGGGCAACTTCACAATACTTTTATCACAgc
UBR5-mut-R	ggccgcTGTGATAAAAGTATTGTGAAGTTGCCCCATCATATGTACAAA AAAAGTGCCCTCACTCAAATGAGCAAATTACc

wt: wild type; mut: mutation; F: sense strand; R:antisense strand. Uppercase letters represent the interest sequence. Lowercase letters represent the sequence used for ligation.

Table S3. Sequences of miR-PC-2869 mimics and siRNAs used for RNA interference

oligonucleotide name	Anti-sense strand (5′-3′)
miR-PC-2869	CGCAUUGGUGGUUCAGUGG
siCDK8	UUUAGAAGCUCUGUGAAACdTdT
Ce-siEEF1A1	UCUUAAUAUAGGUGCUGACUU
Ce-siNTN1	CACGUAGGUCACCUCGAACUU
Hs-siEEF1A1	UUCACUCAAAGCUUCAUGGdTdT
Hs-siNTN1	UCACUUCGAACUUCUUGCCdTdT

siCDK8 is effective in both antler cartilage cells and human cells. Ce: *Cervus elaphus*; Hs: *homo sapiens*.

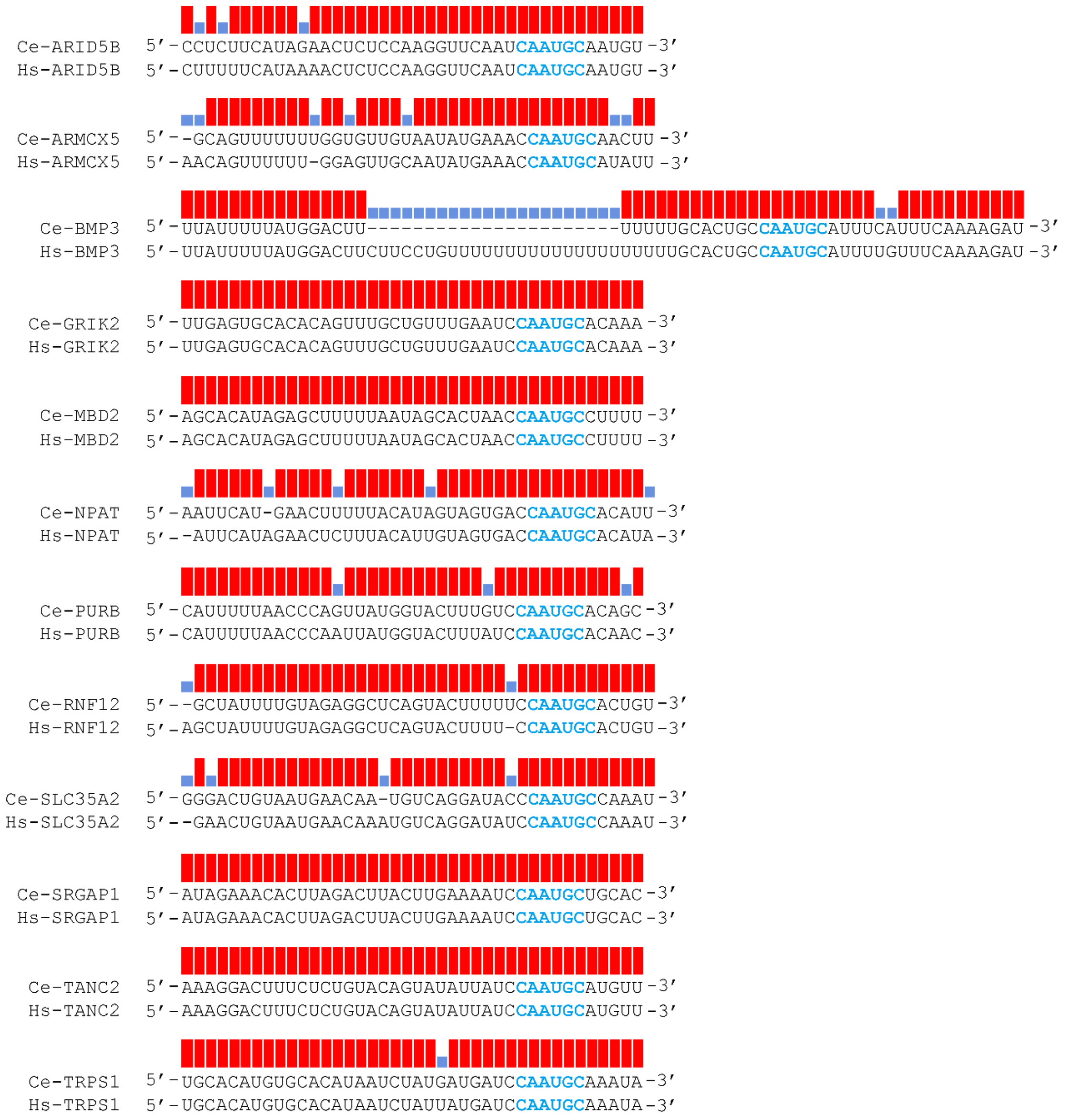

Figure S1. The specific sites recognized by miR-PC-2869 in 12 target genes are conserved between humans and red deer (*Cervus elaphus*). Blue bases represent the sequence complementary to the seed sequence of miR-PC-2869.
